# Evaluation and Classification of Atrial Arrhythmias from Signatures in Electrographic Flow Metrics

**DOI:** 10.19102/icrm.2026.17075

**Published:** 2026-07-15

**Authors:** Steven Castellano, Melissa H Kong, Joshua D’Arcy, Dmytro Perekrestenko, Kent R Nilsson, John D Hummel

**Affiliations:** 1 Cortex, Inc., Menlo Park, CA, USA; 2 Department of Computer Science, Northwestern University, Chicago, IL, USA; 3 Ablacon, Inc., Zurich, Switzerland; 4 Department of Cardiac Electrophysiology, Piedmont Heart Institute, Athens, GA, USA; 5 Department of Cardiac Electrophysiology, Augusta University—University of Georgia, Athens, GA, USA; 6 Department of Cardiology, Ohio State University Wexner Medical Center, Columbus, OH, USA

**Keywords:** Arrhythmias, basket catheter, cardiac, electrographic flow mapping, panoramic mapping

## Abstract

In patients undergoing atrial fibrillation (AF) ablation, it can be difficult at times to distinguish between AF, micro–re-entrant atrial flutters (AFLs), and focal atrial tachycardias (ATs). Understanding the mechanism of arrhythmogenesis can guide ablation lesion sets. Electrographic flow (EGF) mapping displays global atrial wavefront propagations in near-real time. Patterns of EGF can show both regions of stable flow direction over time and disorganized electrical wavefronts. In so doing, it can differentiate mechanisms of arrhythmogenesis. The objective of this study was to evaluate the EGF patterns and metrics that may be used to successfully differentiate mechanisms of arrhythmogenesis. A total of 2933 recordings from 151 patients who underwent EGF mapping were analyzed to determine metrics that could distinguish between mechanisms of arrhythmogenesis. These EGF-derived metrics include: (1) electrographic flow consistency (EGFC), (2) flow angle variability (FAV), (3) source activity, (4) atrial cycle length, (5) mean R–R correlation, and (6) disorganization score. The disorganization score varied the most between AF and AFL or AT (*P* < .0001), while the mean EGFC varied the most between AFL and AT (*P* < .0001). The mean FAV was found to be strongly correlated with both EGFC (*r*^2^ = 0.767; *P* < .0001) and the disorganization score (*r*^2^ = 0.552; *P* < .0001); therefore, the mean FAV was shown to contain the composite information of the other two metrics. A multilayer perceptron trained and tested on an equal number of recordings in each arrhythmia was also able to accurately determine the rhythm from previously unseen samples with 81% accuracy when given only the five remaining metrics. Additionally, it was 100% successful at distinguishing AF from the more organized rhythms. In conclusion, summative EGF metrics that characterize vector flow provide signature patterns of global atrial wavefront propagations in different atrial arrhythmias, which can be used to successfully determine the mechanisms of atrial arrhythmias.

## Introduction

Atrial fibrillation (AF) is a common atrial arrhythmia that can spontaneously convert to organized atrial arrhythmias such as macro–re-entrant atrial flutter (AFL),^1,2^ micro–re-entrant atrial tachycardia (AT)/AFL, or focal AT.^3,4^ Rhythm conversions of AF to other atrial arrhythmias most often occur with anti-arrhythmic medications^5^ or during cardiac ablation.^6–8^ In the latter case, ablation lesions can result in the organization of fibrillatory conduction or create scarring and subsequent tissue anisotropy that alters the arrhythmic substrate. Moreover, organized AF can look similar to AFL and AT, and differentiating these mechanistically can be challenging. Accurately diagnosing the mechanism of arrhythmia can alter ablation lesion sets, thereby improving outcomes and shortening procedure times.

Electrographic flow (EGF) mapping provides data toward this end. EGF maps are computed by applying the Horn–Schunck algorithm to unipolar electrograms (EGMs) recorded from a 64-electrode basket catheter to generate full spatiotemporal reconstructions of atrial electrical wavefront propagation patterns.^9–11^ The distinct activation patterns in these maps have been previously used to differentiate mechanisms of arrhythmias in animal models.^12^

EGF consistency (EGFC), derived from the EGF maps, provides a measure of organization to characterize the flow of electrical activation through the atria.^13^ Regions of coherent flow have a greater EGFC compared to areas of disorganized flow. Accordingly, the EVAL-AF (“Electrographic Flow Mapping and Concomitant Voltage Mapping in Sinus Rhythm and Atrial Fibrillation”) clinical trial demonstrated that patients had a higher EGFC in sinus rhythm versus AF and that EGFC was correlated with bipolar voltage in both rhythms.^14,15^ The EVAL-AF results therefore suggest that EGFC may be useful in differentiating AF from other, more organized rhythms.

EGF maps can also localize extra-pulmonary vein (PV) sources that serve as AF triggers, which are prime candidates for adjunctive focal ablation.^9,16^ These potential target sources are differentiated/identified based on the percent of source activity representing how often they serve as a trigger driving wavefront propagation. From the combination of EGFC and source activity, AF patients have been successfully phenotyped into subgroups with different pathophysiologies, recommended treatments, and likelihoods of recurrence.^17–21^

Several other metrics are also derived from the EGF maps that may aid in understanding circuits and rhythm classification. Chief among these is the flow angle variability (FAV), which measures the change in vector direction between 19-ms frames of a recording. Accordingly, EGFC measures the average magnitude of EGF vectors, while FAV measures their directional variance over time. Other relevant EGF metrics are derived from the EGMs and include the mean atrial cycle length (CL) that measures the average time between atrial depolarizations, an R–R correlation (R–RC) score that shows the extent of relatedness of consecutive R–R intervals, and a disorganization score that quantifies the extent of dissimilarity among all R–R intervals.

The purpose of this investigation is to evaluate the use of the six EGF metrics of EGFC, source activity, FAV, mean atrial CL, R–RC score, and disorganization score in identifying patterns during AF, AFL, and AT. The study further aims to determine the most valuable metrics to classify recordings based on the likely arrhythmia mechanism.

## Methods

### Electrographic flow mapping in atrial arrhythmias

A total of 2933 1-min recordings were analyzed from 151 AF patients who underwent EGF mapping with a 64-pole basket catheter. Recordings were taken from 69 patients who had EGF maps retrospectively generated from FIRMap™ EGMs^16^ and from 82 prospective patients who were mapped in the FLOW-AF (“A Randomized Controlled Study to Evaluate the Reliability of the Ablacon Electrographic FLOW [EGF] Algorithm Technology [Ablamap Software] to Identify AF Sources and Guide Ablation Therapy in Patients with Persistent Atrial Fibrillation”) randomized clinical trial.^17^ All clinical data were collected from clinical trials performed in line with the principles of the Declaration of Helsinki. Ethics approval was obtained from the ethics committees of participating institutions. Informed consent was obtained from all individual participants included in the study.

EGF mapping was performed using the Optimap^®^ software (Ablacon, Wheat Ridge, CO, USA). Unipolar EGMs were recorded using a commercially available 64-electrode basket mapping catheter (FIRMap™; Abbott, Chicago, IL, USA) connected to a proprietary, CE-marked recording system (EP Map™; EPMap-System GmbH, Herdecke, Germany). Each recording was 1 min long, and the unipolar signals were processed using a proprietary algorithm to remove the QRS complex, noise, baseline fluctuations, and far-field signals. Using a biharmonic spline interpolation based on Green’s function, the electrical field was estimated, and a Horn–Schunck flow estimation of the Green’s interpolation frames was subsequently performed to visualize atrial wavefront propagation over time. An in-depth explanation of the theoretical basis for EGF mapping and details of the EGF mapping algorithm were previously published by Haines et al.^9^ and summarized by Castellano and Kong.^10^ The dominant patterns of wavefront propagation over time were displayed in flow vector maps, enabling the visualization of origins of excitation representing extra-PV AF triggers, called sources. Active sources exhibited divergent flow fields.^22^

In most cases, EGF mapping was obtained from the 64-pole basket catheter placed in multiple standardized positions within each atrium. There were two standardized positions in the left atrium and three standardized basket positions in the right atrium. Mapping in additional basket positions was also performed if necessary to obtain complete endocardial atrial coverage. Moreover, repeat recordings in the same positions were often employed to assess local changes after PV isolation or source ablation.

### Rhythm determination from electrophysiological data

Rhythms were determined from EGMs and associated electrophysiological data by the operating physician concurrently with each map generation. Before a rhythm determination was made, activation patterns and CLs of the EGMs recorded by the 64-basket mapping were analyzed.

Overall classification was based on prespecified electrophysiologic criteria. AF was characterized by the absence of a consistent global activation sequence, beat-to-beat variability in CL (>20% variation), spatially disorganized activation across splines, and the presence of continuous or highly fractionated EGMs. AFL was characterized by a stable atrial CL (<10% variation), sequential activation consistent with macro–re-entry across the basket, continuous activation spanning the entire CL without electrical quiescence, and evidence of a consistent propagation direction across beats. AT was characterized by a discrete focal earliest activation site with centrifugal spread, a stable CL, the presence of an isoelectric interval between activations in a majority of electrodes, and the absence of a complete macro–re-entrant activation loop.

When rhythm classification remained uncertain, additional maneuvers (entrainment pacing or adenosine administration) were performed at the operator’s discretion and incorporated into the final adjudication.

### Computing electrographic flow consistency, flow angle variability, and source activity from electrographic flow maps

Overall, EGFC measured the net magnitude of wavefront propagation vectors over time, FAV measured the rate of angle change in the vectors, and source activity measured the percentage of time a source was on.

EGFC was computed from the Euclidean length of the localized vector fields, and its mean provided a measure of the overall magnitude of flow averaged over all basket electrodes and all 60 s of recording time. Notably, because approximately 30% of electrodes of the spherical basket catheter are floating in space in each standardized mapping position, electrodes with low contact were removed from the mean EGFC calculation due to the presence of artificially low local EGFC in the locations of non-contacting electrodes. The methodology for this automated subtraction has been described previously by Nilsson et al.^19^

FAV was calculated as the number of degrees the flow vector angles change between consecutive 19-ms frames of the EGF maps. Its mean is the average of the frame-to-frame angle deviations over the duration of an entire recording.

Source activity was quantified as the percentage of 2-s segments within a recording during which the dominant source was emanating flow. For activity to be considered as originating from a single source, the location could not vary by more than one electrode distance (approximately 2 cm) throughout the 60-s recording.

### Developing an R–R correlation score and disorganization score from electrograms

Overall, the R–RC score measured the consistency of consecutive R–R intervals, while the disorganization score measured the inconsistency of all R–R intervals.

To establish the R–RC score, the R–R intervals in the individual EGMs were first isolated from all 64 electrodes, and their durations were measured. The distribution of R–R intervals was approximated via a Gaussian mixture fitting, where the centers of up to four Gaussians were initialized with the k-means method. The R–R intervals that appeared within ±1 standard deviation of the mean of the narrowest Gaussian were pulled, and the Pearson correlation coefficient (*r*) of each consecutive pair of intervals per channel was computed. The mean *r* over the top 5% of pairs was then labeled the mean R–RC score of the recording. This score was calculated from only 5% of the most related R–R intervals for the purposes of removing noise and discriminating among the most organized segments of each recording.

A disorganization score was also developed to account for instances where related R–R intervals were not consecutive. To do so, the time shift that produced the highest mean R–RC over all channels was first calculated, and the *r* for each pair of intervals per channel before versus after the time shift was computed. The mean *r* over the top 5% of time-shifted pairs was then labeled the mean R–R cross-correlation (R–RXC) score, and the disorganization score was determined as 1 − max(mean R–RC score, mean R–RXC score). Accordingly, the disorganization score was designed to be inversely related to R–RC and to additionally account for instances of time-shifted relatedness of R–R intervals.

### Building the neural network

A multilayer perceptron (MLP) neural network was built in TensorFlow (Google LLC, Mountain View, CA, USA), employing the metrics deemed most relevant in orthogonally differentiating the three atrial arrhythmias. The input metrics were passed through a four-layer neural network with two hidden layers of 81 and 27 units, respectively. The network was trained with exponential linear unit activation functions, a categorical cross-entropy loss function, and an Adam optimization algorithm. The output layer leveraged a softmax activation function. Five-fold cross-validation was employed such that 64% of the input EGF metric arrays were used for training, 16% were used for validation, and the remaining 20% were used for testing and determining prediction accuracy. Fifty-two samples in each rhythm were randomly selected such that there were 156 total samples prior to splitting.

Thus, in simple terms, a fundamental form of classifier neural network—namely, an MLP—was trained to recognize patterns in the metrics described in the previous sections for the purposes of classifying the heart rhythm as AF, AFL, or AT. The model learned from a portion of the data and was then tested on separate, unseen samples to estimate how accurately it would perform in practice.

### Statistical analysis

Summary data were expressed as mean value ± 1 standard deviation. *P* values for group comparisons were computed using two-tailed, independent *t* tests for continuous variables. Linear regression was calculated using a line of best fit, and the coefficient of determination (*r*^2^) and *P* value for the F-test of significance of the slope were reported. For all statistical tests, the null hypothesis was rejected at the level of *P* < .05.

## Results

### Patient demographics

Overall, 2933 recordings were taken from 151 patients, 17 (11%) of whom underwent spontaneous rhythm changes from one atrial arrhythmia to another mid-procedure. There were 2718 AF recordings from 150 patients, 53 (35%) of whom were female, with a weighted mean age of 64.6 ± 9.4 years and a left atrial diameter (LAD) of 4.72 ± 0.57 cm. In addition, there were 52 AFL recordings from 10 patients, four (40%) of whom were female, with a weighted mean age of 67.8 ± 6.0 years and an LAD of 4.20 ± 0.45 cm. Finally, there were 163 AT recordings from 18 patients, seven (39%) of whom were female, with a weighted mean age of 57.8 ± 9.7 years and LAD of 3.40 ± 0.45 cm.

### Exploring patterns in atrial arrhythmia from electrographic flow metrics

From looking at sample EGF maps and their constituent EGMs, clear differences were generally apparent among the different arrhythmias, as shown in **Figure 1**. Patients in AF had generally more chaotic wavefront patterns, resulting in visibly lower EGFC in each frame and higher FAV between frames versus the more organized arrhythmias. Sources were also less likely to turn off in AFL and AT, often creating higher activity than in AF. Additionally, the EGMs showed lower minimums in mean atrial CL, as well as longer and more variable R–R intervals in AF versus AFL and AT.

**Figure 1:**
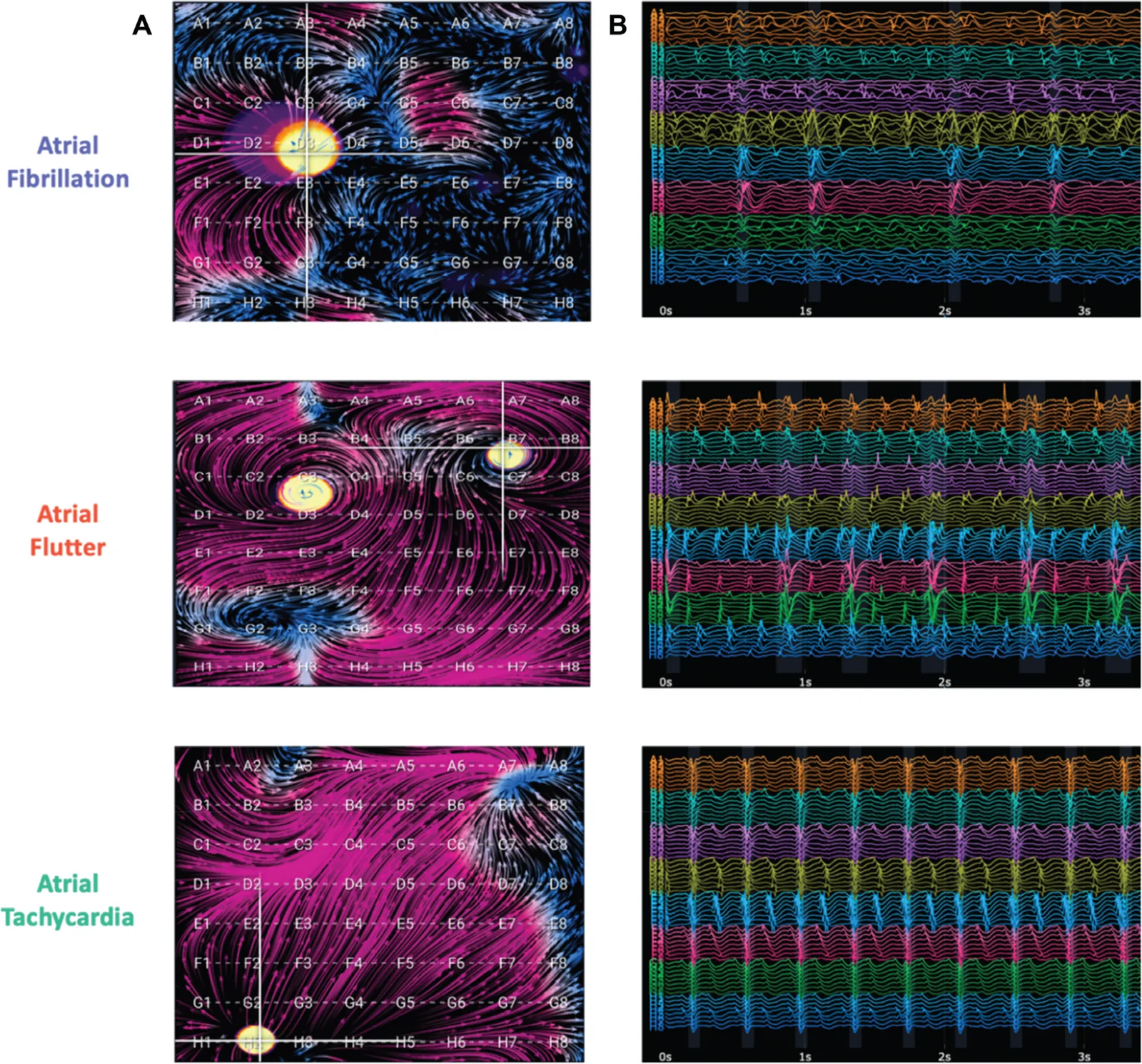
Electrographic flow (EGF) and electrogram (EGM) rhythm differences. **A:** Example EGF maps in three atrial arrhythmias. In the atrial fibrillation (AF) recording, there are chaotic wavefront collisions and more areas of low EGF consistency (EGFC) (blue) versus high EGFC (purple). An active source at D3 emits flow near the broadest patch of high EGFC. In the atrial flutter (AFL) recording, there are more organized high EGFC areas versus low EGFC areas. The active source at B7–C7 emits flow in all directions, and there is re-entry around the C3–D3 passive rotor. In the atrial tachycardia (AT) recording, flow diverges from a focal source with broadly high EGFC creating organized but non–re-entrant circuits. **B:** Respective EGMs of the EGF maps in **A**. In the AF EGM, fibrillatory waves are apparent, and the R–R interval length is variable, yielding a low mean R–RC score and a high disorganization score. In the AFL EGM, there are organized P-waves and some related but non-consecutive R–R intervals, such as at 1.3–1.9 s and 2.6–3.2 s, yielding a low mean R–RC score but a low disorganization score. In the AT EGM, there are organized P-waves and R–R intervals of a consistent 0.4-s length, yielding a high mean R–RC score and a low disorganization score.

Thus, several metrics were computed from the 2933 available recordings, and their means and standard deviations were calculated for each rhythm, as shown in **Table 1**. AF recordings had lower mean EGFC, activity, mean atrial CLs, and mean R–RC scores but had higher mean FAV and disorganization scores than AFL or AT recordings (*P* < .0001). Separately, AFL recordings had higher mean EGFC (*P* < .0001) and atrial CLs (*P* < .0001) but lower mean FAV (*P* < .0001) than AT recordings. The lowest *P* values for AF versus AFL and AF versus AT were in the disorganization score, and the lowest *P* value for AFL versus AT was in the mean EGFC.

**Table 1:** EGF Metric Differences in Three Atrial Arrhythmias

	AF	AFL	AT	AF vs. AFL *P* Value	AF vs. AT *P* Value	AFL vs. AT *P* Value
Mean EGFC (AU)	0.586 ± 0.241	1.125 ± 0.150	0.879 ± 0.287	4.37 × 10^−32^	8.55 × 10^−27^	2.22 × 10^−13^
Mean FAV (°/19 ms)	3.53 ± 1.02	1.47 ± 0.54	2.01 ± 0.87	5.37 × 10^−34^	8.31 × 10^−52^	5.20 × 10^−7^
Activity (%)	24.5 ± 14.0	43.0 ± 27.6	45.8 ± 29.6	1.50 × 10^−5^	3.06 × 10^−16^	.541
Mean atrial CL (ms)	172 ± 20	194 ± 26	178 ± 25	1.25 × 10^−7^	2.59 × 10^−3^	2.03 × 10^−4^
Mean R–RC score	0.510 ± 0.174	0.839 ± 0.138	0.851 ± 0.164	5.17 × 10^−23^	9.16 × 10^−63^	.601
Disorganization score	0.259 ± 0.150	0.019 ± 0.014	0.026 ± 0.043	5.32 × 10^−235^	6.22 × 10^−196^	.0545

*Abbreviations:* AF, atrial fibrillation; AFL, atrial flutter; AT, atrial tachycardia; CL, cycle length; EGFC, electrographic flow consistency; EGM, electrogram; FAV, flow angle variability; R–RC, R–R correlation.

Plots were also made to display the overall scatter and distribution of the core metrics, as shown in **Figure 2**. Distributions were generally non-skewed but non-normal, at times showing outliers or bimodality. No rhythm pairs had entirely non-overlapping distributions for any single metric, but the center of the distribution for AF was often readily distinct from that of AFL or AT.

**Figure 2:**
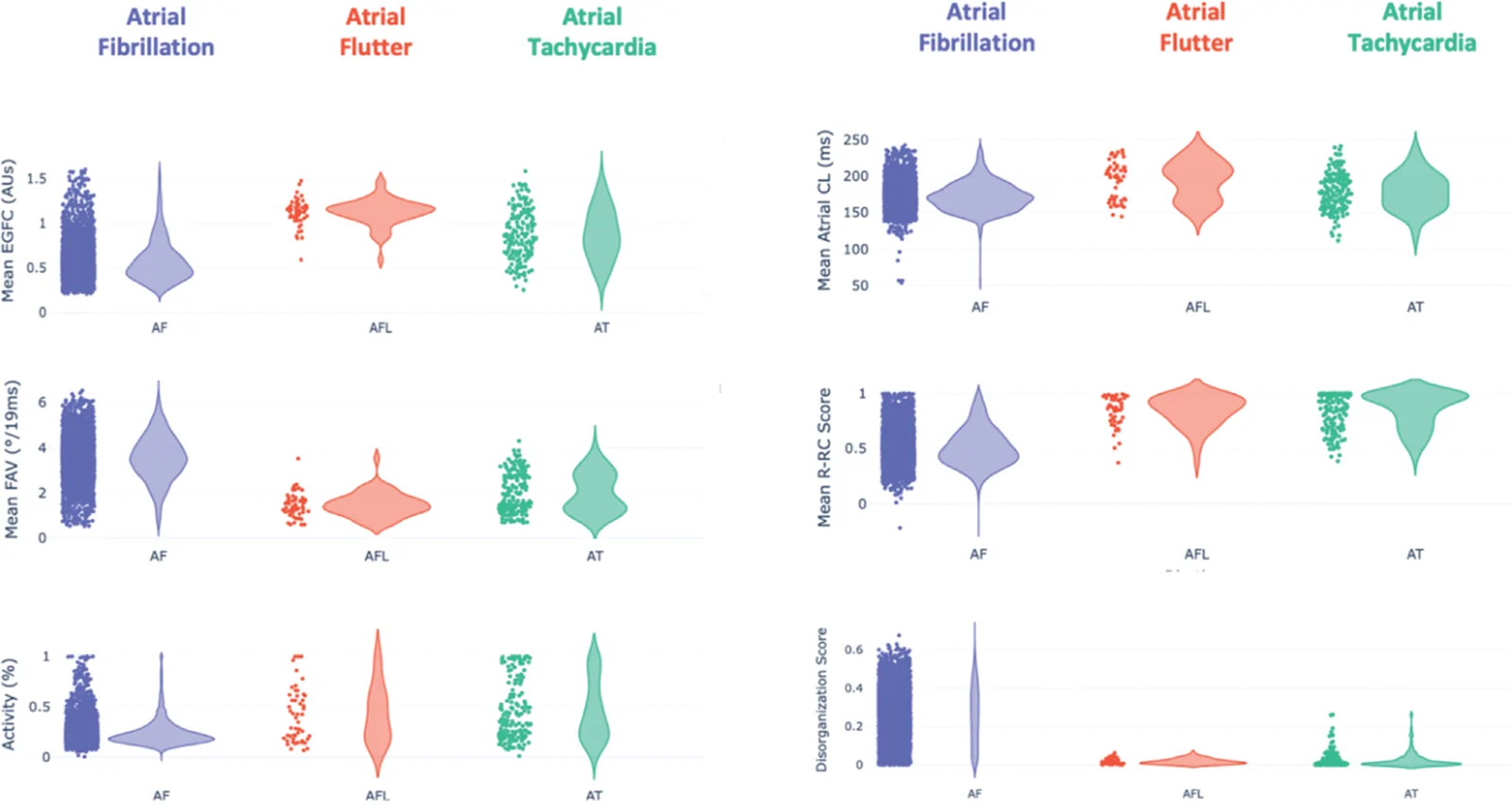
Electrographic flow (EGF) metric distribution by rhythm. Scatter and shape of the distribution are shown. Map metrics are shown in the left column with lower EGF consistency (EGFC) and activity in atrial fibrillation (AF) versus atrial flutter (AFL) and atrial tachycardia (AT) but higher flow angle variability (FAV). Electrogram (EGM) metrics are shown in the right column with a lower mean atrial cycle length (CL) in AF and AT versus AFL, lower mean R–R correlation (R–RC) score in AF versus AFL and AT, and higher disorganization score in AF versus AFL and AT. Distributions are often non-normal.

### Determining relationships among key metrics

A plot of every pair of EGF metrics was generated to determine the relatedness of these rhythm-differentiating variables, as shown in **Figure 3A**. Most metrics demonstrated independence from one another, with only three metric pairs having an *r*^2^ > 0.5, as follows: mean FAV and mean EGFC (*r*^2^ = 0.767; *P* < .0001), mean R–RC score and disorganization score (*r*^2^ = 0.615; *P* < .0001), and mean FAV and disorganization score (*r*^2^ = 0.552; *P* < .0001), as detailed in **Figure 3B**. The next strongest relationships were noted between mean FAV and mean R–RC score (*r*^2^ = 0.466; *P* < .0001), mean EGFC and mean R–RC score (*r*^2^ = 0.426; *P* < .0001), and mean EGFC and disorganization score (*r*^2^ = 0.391; *P* < .0001), respectively. All other metric pairs had *r*^2^ < 0.15. No meaningful relationship among any of the EGF metrics was established with demographic metrics, including patient age, LAD, and left ventricular ejection fraction (*r*^2^ < 0.01; *P* > .05), although there was a weak relationship between mean atrial CL and age (*r*^2^ = 0.099; *P* < .0001).

**Figure 3:**
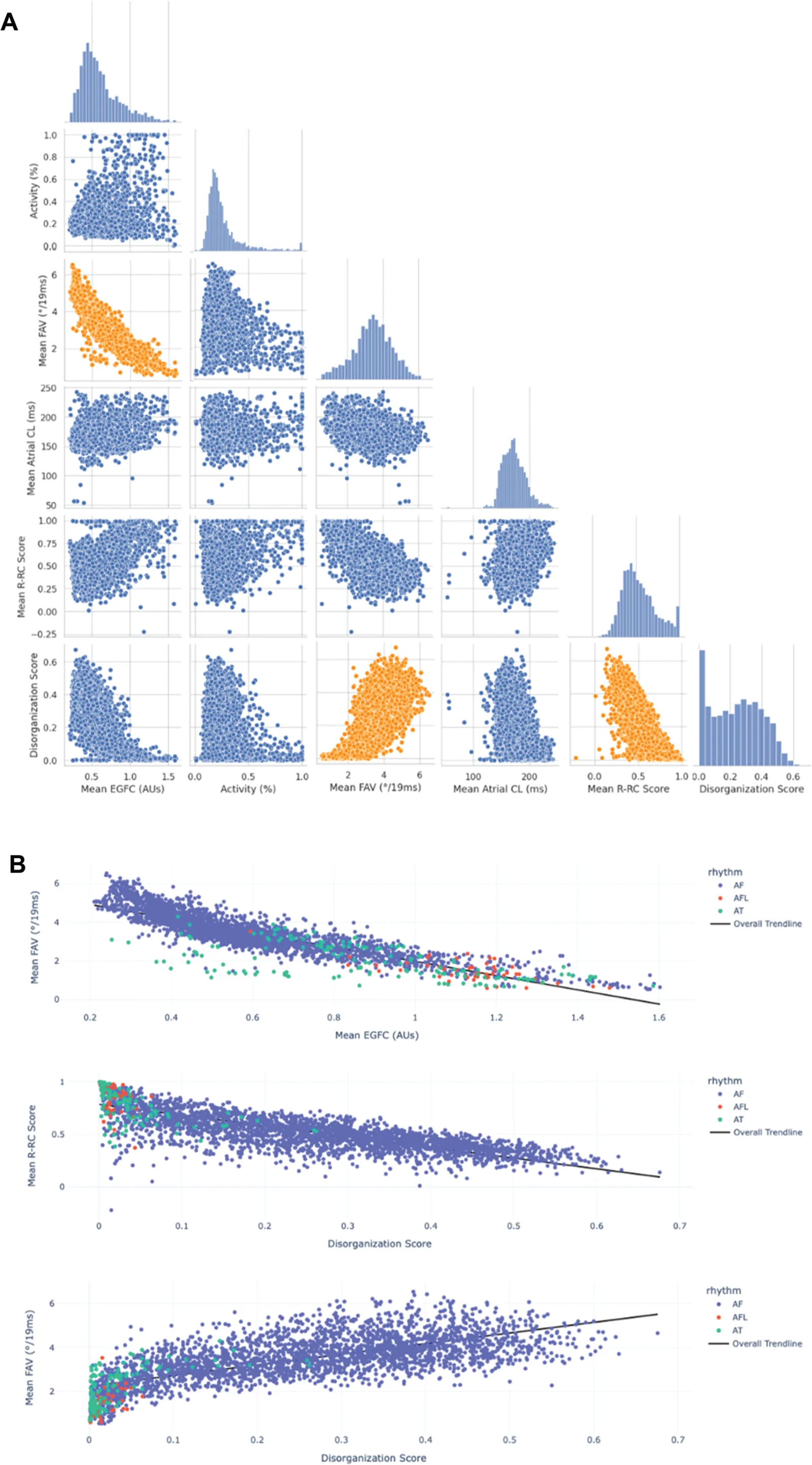
Electrographic flow (EGF) metric relationships. **A:** Plots of every pair of metrics under study. The distribution of the individual metrics is shown above that metric along the diagonal. Plots with *r*^2^ > 0.5 are highlighted. **B:** Overall trendlines of the highlighted three most correlated metric pairs from **A**, colored by rhythm: mean flow angle variability (FAV) versus mean EGF consistency (EGFC) (*r*^2^ = 0.767; *P* < .0001), mean R–R correlation (R–RC) score versus disorganization score (*r*^2^ = 0.615; *P* < .0001), and mean FAV versus disorganization score (*r*^2^ = 0.552; *P* < .0001). *Abbreviations:* AF, atrial fibrillation; AFL, atrial flutter; AT, atrial tachycardia; CL, cycle length.

### Employing a multilayer perceptron to classify atrial arrhythmias from summative electrographic flow metrics

An MLP was constructed to use these EGF metrics to understand patterns in the three studied atrial arrhythmias and then classify previously unseen metric sets based on the most likely arrhythmia mechanism into which they fell. Before doing so, 52 recordings were randomly selected in each arrhythmia to be used for training and testing the MLP. An equal number of recordings in each rhythm were pulled so as not to make the task trivial due to the preponderance of recordings in AF creating a large class imbalance. Additionally, the mean FAV of the recording was not given to the MLP because its strong correlations with both mean EGFC and disorganization score implied that mean FAV would add redundancy to the model. Therefore, only five summative metrics were used for training and testing the model: mean EGFC, source activity, mean atrial CL, mean R–RC score, and disorganization score.

The MLP was 81% accurate at predicting the atrial arrhythmia from the testing set of EGF metrics. AF was classified with 100% accuracy. A receiver operating characteristic (ROC) curve for the one-versus-rest classification of each rhythm, as well as the micro-averaged and macro-averaged ROC curves for these classifications among all three rhythms, is shown in **Figure 4A**. AF had an area under the ROC curve (AUC) of 100%, AFL had an AUC of 94% ± 3%, and AT had an AUC of 82% ± 5%. An accompanying principal component analysis (PCA) plot is shown in **Figure 4B**. AF was again completely separable from AFL and AT despite some seemingly related edge cases, while AF and AT were largely distinct but contained an area of significant overlap.

**Figure 4:**
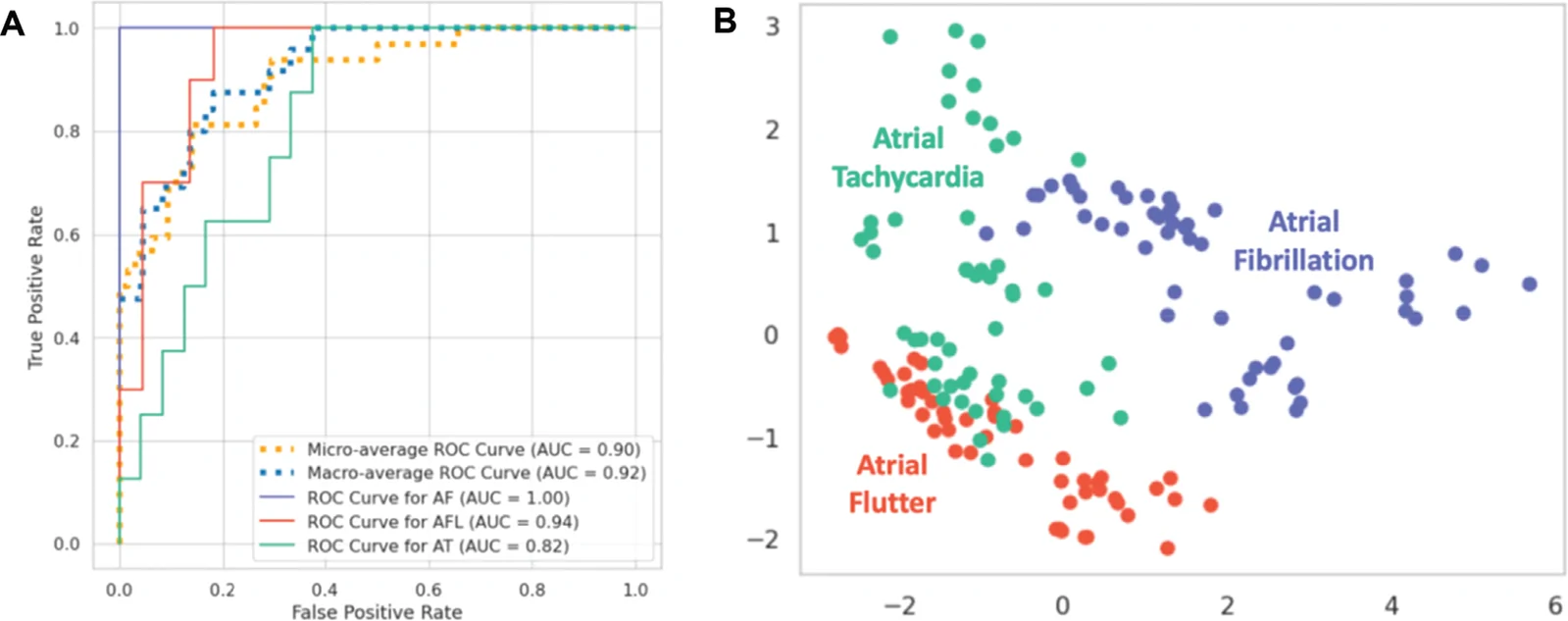
Convolutional neural network (CNN) performance. **A:** Receiver operating characteristic (ROC) curve of the micro-averaged, macro-averaged, and one-versus-rest multiclass predictions for each rhythm. Atrial fibrillation (AF) is 100% separable from the organized rhythms. Area under the ROC curve (AUC) for each curve is shown in the key. **B:** Two-dimensional principal component analysis plot of all 156 recordings used to train and test the CNN. AF is non-overlapping with atrial flutter and atrial tachycardia.

## Discussion

The data from prior clinical trials that leveraged EGF mapping were analyzed to understand differences in EGF metrics among the most frequently seen atrial arrhythmias. The key findings are as follows:

1. Six EGF metrics were identified that were significantly different between AF and both AFL and AT, three of which were also significantly different between AFL and AT.
2. Mean FAV was found to contain the composite of the information in the mean EGFC and disorganization score.
3. An MLP was built that could classify equal samples of AF, AFL, and AT with 81% accuracy, including 100% accuracy at distinguishing AF.

Collectively, these findings show that the analyzed arrhythmias behave in clusters of distinct patterns that can be captured from the summative metrics found in EGF maps.

From the analysis of individual metrics, patterns arose between the analyzed atrial arrhythmias, but no single metric could fully differentiate any of the rhythms from others. Such a pattern likely exists because all three arrhythmias have variegated mechanisms and presentations. In AF, the mean EGFC was lowest on account of the chaotic wavefronts creating turbulent and colliding vectors with low magnitudes. Moreover, this finding is consistent with EGFC serving as a measure of atrial health related to bipolar voltage. The mean FAV was accordingly highest in AF because larger frame-to-frame variance in vector direction resulted from the more turbulent wavefronts that already had a lower baseline magnitude. Source activity was then lowest in AF due to sources being most prone to turn off in this more disorganized rhythm. In the EGMs, the mean atrial CL was lowest in AF in accordance with predicate data.^23,24^ The R–R intervals were also the most inconsistent, yielding lower mean R–RC scores and much higher disorganization scores.

When contrasting AFL and AT, the mean EGFC was higher and the mean FAV was lower in AFL. This is so likely because AFL represents a re-entrant circuit with high-magnitude flow in a consistent direction around a region of passively activated tissue, while the presentation of AT is more variable. The mean atrial CL was also higher in AFL but with two modes, which may result from typical and atypical AFL having different CLs.^25^ Activity, mean R–RC score, and disorganization score were not significantly different between AFL and AT, indicating that fluctuations in focal triggers and R–R intervals are largely similar between the two rhythms. Distributions of AFL and AT were also non-skewed but occasionally bimodal, suggesting that there may be clusters of two or more distinct arrhythmia presentations based on the mechanistic subtype of AFL or AT. One example of this phenomenon is in the aforementioned cases of typical and atypical AFL.

When assessing the metrics for inter-metric correlations, three of the 15 metric pairs had *r*^2^ > 0.50, indicating that >50% of the internal variance in one of the metrics could be accounted for by the interval variance in the other. Least surprising of these pairs was the (1) mean R–RC score and disorganization score (*r*^2^ = 0.615), as the disorganization score was partially derived from the mean R–RC score. The other high correlations were between (2) mean EGFC and mean FAV (*r*^2^ = 0.767) and (3) disorganization score and mean FAV (*r*^2^ = 0.552). Both relationships are also intuitive: for (2), as vector magnitudes decrease, their direction is more readily changed, and, for (3), as ventricular depolarization rates vary over time, so too would the directions of flow patterns through the atria that drive these depolarizations.

It should also be noted that the mean R–RC score correlated with the mean FAV (*r*^2^ = 0.466) and the mean EGFC (*r*^2^ = 0.426), just not as strongly as either of these variables correlated with the disorganization score. Beyond these four metrics were activity and mean atrial CL, which were largely independent from the other studied metrics (*r*^2^ < 0.15). After all, active sources can drive chaotic or organized flow patterns, so activity could either wax or wane as atrial health decreases. CL is also not directly associated with specific flow patterns or the relatedness of R–R intervals.

Collectively, an MLP built using the five metrics of mean EGFC, activity, mean atrial CL, mean R–RC score, and disorganization score was able to classify previously unseen, equally sized samples of the three atrial arrhythmias with 81% accuracy. Such strong performance from just five pieces of summative data is notable, especially when considering that AF was identified with 100% accuracy.

ROC curves are graphical tools that show how well a model distinguishes between categories by illustrating the trade-off between correctly identified cases and false alarms (false positives) across different decision thresholds. From the ROC curves for the MLP, false positives accumulated more rapidly when labeling AT than AFL, which reinforced that AT exists in a space between the generally disorganized AF and the highly organized AFL, though it is more closely tied to AFL. Moreover, PCA plots display complex datasets in a simplified visual space where points that appear close together have similar underlying features, allowing natural groupings into clusters, including visualization of where overlap between clusters occurs. The PCA plot thus depicted that there is a spectrum from AF to AT to AFL: there is full separability of AF from both AFL and AT that can be readily visualized with AFL further than AT, but there also appears to be a close association between a subtype of AFL and a subtype of AT.

These findings are compelling not only for what they reveal about the EGF properties of atrial arrhythmias but also in their applications for patient management. For example, future recordings can be characterized based on where they fall within the PCA map clusters, or the studied EGF metrics can be employed more generally to generate an arrhythmia organization score. Gathering such a score for each patient at baseline and then measuring any changes over procedure time can make it easier to notice rhythm changes in future patients. Such a score would also allow physicians to see if patients are moving toward a more organized arrhythmia after each ablation more readily than they can from monitoring the unipolar EGMs or even from tracking changes in the processed EGF metrics. Endeavors to characterize EGF rhythms from recordings of even a single atrial cycle are accordingly under further development.^26^

Ultimately, we built a rhythm classifier that could use a low amount of data—just five EGF metrics—to classify an atrial arrhythmia with high accuracy. We further show that EGFC and activity, which are the two metrics employed to characterize patients’ AF phenotypes and consequent post-procedure freedom from AF, are also the primary metrics used to classify patients’ rhythms. We therefore believe that monitoring both the magnitude of EGF flow vectors (EGFC) and the on-time of EGF sources (source activity) is paramount for understanding atrial wavefront propagation patterns regardless of rhythm. EGF metrics can therefore be used to characterize arrhythmias pre- and post-procedure or between repeat procedures. The metrics can further monitor mid-procedure response to ablation, including readily revealing progression toward rhythm organization even when this progression does not itself result in a rhythm change. Such tracking would be useful both in guiding therapy toward treating the appropriate arrhythmia and in monitoring response to ablation.

### Limitations

The major limitation of this study is the relatively low number of patients and recordings for each type of arrhythmia mechanisms, particularly AFL and AT, given the wide variability in how these rhythms can present. Although AF patients were the most numerous, they predominantly had recurrent or persistent AF, which is perhaps easier to distinguish from AFL and AT than paroxysmal AF due to its generally more advanced pathophysiology. Instances of AFL and AT were likewise limited to those that arose in AF patients. Other atrial arrhythmias, eg, atrial premature complexes and multifocal AT, were also excluded from this analysis.

### Future directions

Based on the findings of this study, we aim to identify and study additional patients who undergo rhythm changes characterized by organization or disorganization into different arrhythmias during an ablation procedure. Using baseline signatures in the EGF metrics from AF recordings as a starting point, we can track the level of organization throughout a procedure. We also seek to understand how the studied metrics relate to atrial health and one’s individual mechanism of disease in further developing personalized, targeted treatments for AF.

## Conclusions

Using an MLP based on six EGF metrics, atrial arrhythmias can be accurately classified based on arrhythmia mechanism. Collectively, these data allow us to better classify rhythm changes in AF patients and to build upon our understanding of the EGF metrics that are derived from endocardial recordings. While EGFC and activity have already demonstrated importance in classifying mechanisms of AF, they now provide additional relevance in identifying signature patterns among related atrial arrhythmias.
